# Reduction in COVID-19 related resource loss and decline in prevalence of probable depression in Chinese adults: an application of the Conservation of Resource Theory

**DOI:** 10.1186/s40249-023-01068-1

**Published:** 2023-03-16

**Authors:** Yanqiu Yu, Mason M. C. Lau, Joseph T. F. Lau

**Affiliations:** 1 Department of Preventive Medicine and Health Education, School of Public Health, Fudan University, Shanghai, China; 2 Key Laboratory of Public Health Safety, Fudan University, Shanghai, China; 3 Centre for Health Behaviours Research, Jockey Club School of Public Health and Primary Care, The Chinese University of Hong Kong, Hong Kong, China; 4 Zhejiang Provincial Clinical Research Center for Mental Disorders, The Affiliated Wenzhou Kangning Hospital, Wenzhou Medical University, Wenzhou, China; 5 School of Mental Health, Wenzhou Medical University, Wenzhou, China; 6 School of Public Health, Zhejiang University, Hangzhou, China

**Keywords:** Depression, Resource loss, COVID-19, Conservation of Resource Theory, Serial cross-sectional study

## Abstract

**Background:**

The levels of resource losses due to coronavirus disease 2019 (COVID-19) and mental distress may change during the pandemic period. Based on the Conservation of Resource (COR) Theory, this study investigated such changes and the mediation between survey time (Round 2 versus Round 1) and depression via resource losses.

**Methods:**

Two serial random population-based telephone surveys interviewed 209 and 458 Hong Kong Chinese adults in April 2020 and May 2021, respectively. Probable depression was defined as 9-item Patient Health Questionnaire (PHQ-9) score ≥ 10. The validated Conservation of Resources Scale for COVID-19 (CORS-COVID-19) scale was used to assess resource losses due to COVID-19. Multivariable logistic regression analysis, hierarchical logistic regression analysis, and structural equation modeling (SEM) was conducted to test the association, interaction, and mediation hypotheses, respectively.

**Results:**

The prevalence of probable depression declined from 8.6% to 1.0% over time, together with reductions in losses of financial resource (Cohen’s *d* = 0.88), future control (Cohen’s *d* = 0.39), social resource (Cohen’s *d* = 0.60), and family resource (Cohen’s *d* = 0.36) due to COVID-19. All the overall scale/subscales of the CORS-COVID-19 were positively and associated with probable depression [adjusted odds ratio (a*OR*) ranged from 2.72 to 42.30]. In SEM, the survey time was negatively associated with the latent variable of resource loss (*β* = − 0.46), which in turn was positively associated with probable depression (*β* = 0.73). In addition, the direct effect of survey time on probable depression was statistically non-significant (*β* = − 0.08), indicating a full mediation effect of resource losses.

**Conclusions:**

The lessening of the resource losses might have fully accounted for the significant decline in probable depression from Month 3 to 15 since the first COVID-19 outbreak in Hong Kong, China. The level of depression might have increased during the first phase of the pandemic, but might decline in the later phases if resources losses could be lessened. All stakeholders should hence work together to minimize individuals’ COVID-19-related resource losses to prevent depression in the general population, as COVID-19 might be lasting.

**Supplementary Information:**

The online version contains supplementary material available at 10.1186/s40249-023-01068-1.

## Background

The coronavirus disease 2019 (COVID-19) pandemic has globally accumulated over 672 million cases and 6.8 million deaths as of February 8, 2023) [1]. Prevalent mental distress have been reported across populations/countries throughout the pandemic period [2]. Temporal trends are expected as the situations keep changing but the direction is unclear, as increasing [3, 4], declining [5, 6], and stable [7] trends of depression/anxiety have been reported prior to the rollout of COVID-19 vaccination. A dearth of studies compared changes in mental distress at population level within the post-rollout periods, or between periods of strict and loose COVID-19 control measures. No study has explained why temporal differences had occurred by testing mediation mechanisms. To reveal changes of the pandemic’s impacts on population mental distress, such research is warranted.

The unprecedentedly strict COVID-19 control measures (e.g., lockdown, travel restrictions, and quarantine) have caused serious damages to almost all aspects of life, resulting in severe losses in resources (e.g., financial resources) that elevated mental distress [8]. The Conservation of Resource (COR) theory was used to provide a dynamic framework of the present study that looked at changes in resource losses due to COVID-19 and depression over time. The theory postulates that everyone is concerned about and motivated to conserve resources as resources mean things highly valued by people; stress would hence arise whenever resource losses have occurred or may occur [9–12]. It posits that resource losses in personal, interpersonal, and material domains due to stressful events (e.g., the COVID-19 pandemic) would cause negative health outcomes, such as mental distress [8]. The theory has been applied to investigate mental health consequences of social changes [13, 14] and natural disasters [15, 16].

Actual and perceived resource losses due to COVID-19 are global and comprehensive, and were closely related to various control measures. Extensive social distancing policies may induce substantial personal (e.g., finance) and interpersonal (e.g., social and family relationships) losses [17]. Closure of entertainment venues and restrictions for traveling would elevate the level of boredom. In Hong Kong, China where the study was conducted, resource losses in the domains of social relationship (e.g., social isolation) [18] and financial resources (e.g., income) [19] have been documented. The Conservation of Resources Scale for COVID-19 (CORS-COVID-19) has recently been developed and validated among Chinese adults [20]. It allows researchers to gauge the levels/changes regarding losses due to COVID-19 in five domains (financial resource, family resource, future control, fun, and social resource). It was associated with emotional distress related to COVID-19 (i.e., panic, anxiety, and emotional agitation). However, the association between resource losses and depression has not been tested. Surveillance of changes in depression over the pandemic period is warranted to inform health promotion and decision making. This has commonly been achieved by serial cross-sectional surveys [21, 22]. The present study was the first one comparing changes in the levels of resource losses due to COVID-19 and depression during the pandemic period.

The present study investigated changes in prevalence of probable depression and levels of resource losses related to COVID-19 over a 12-month period through two serial surveys representing a more severe phase and a recovery phase of the pandemic in Hong Kong, China. It was hypothesized that both the levels of the five domains of resource losses and probable depression would have declined in the general population over time. Second, guided by the COR theory, this study tested the hypotheses whether the five domains of resource losses would be positively associated with probable depression, and whether the strength of such associations would change over time (interaction effects). Third, for the first time, it tested the hypothesis whether the changes in resource losses over time would partially or fully mediate the observed difference in prevalence of probable depression over time.

## Methods

### Participants and data collection

Two serial random telephone surveys were conducted in the adult general population in Hong Kong, China over a 12-month period (Round 1: April 3–10th, 2020; Round 2: May 14–27th, 2021). Inclusion criteria included: (1) those aged 18–70, (2) Hong Kong identity card holders, and (3) Chinese speaking; 209 and 458 participants aged 18‒70 years were interviewed in the two comparable surveys, respectively.

The first survey was conducted during the initial phase of the pandemic and prior to vaccination rollout when class suspension, closure of governmental services, and working from home policies were exercised in Hong Kong, China (January 2020 to January 2021); other policies included social distancing measures (closure of entertainment venues, cancellation of events, and restriction of gathering size ≤ 4 people in all public venues), compulsory facemask wearing in public areas, and boundary control (quarantines for all people entering Hong Kong, China). The economy was then badly hit, partly due to the drop in the number of foreign visitors and social distancing; the local unemployment rate increased from 2.8% in January 2019 (pre-COVID-19 period) to 6.2% in April 2020 (when the first survey was conducted) [23]. Studies conducted around that time found that the social distancing policies were associated with depression in the general population [17, 24].

The second survey was conducted during May 14‒27, 2021, when some control measures were loosened and when vaccination had rolled out since February 26, 2021. The government spent over Hong Kong dollar (HKD) 190 billion [about United States Dollar (USD) 25 billion] supporting affected industries and workers and offered cash rebate of HKD 10,000 (about USD 1300) to all Hong Kong permanent residents in late 2020, which fueled the recovery. The number of reported COVID-19 cases declined from a daily average of 22 to one during the two survey periods. The unemployment rate dropped from the peak of 7.2% in February 2021 to 5.0% in May 2021 [23]; the local gross domestic product (GDP) increased from − 9.1% in the first season of 2020 to 7.2% in the second season of 2021 [25]. Citizens returning from mainland China were waived from quarantine. Restaurants started operating for longer hours. Schools partially resumed. Life in Hong Kong, China was hence returning to ‘normal’. The setting offers a natural ‘social experiment’ involving lowering of resource losses due to COVID-19.

Telephone numbers were randomly drawn from the most updated residential telephone directory; the last two digits were randomized to include some potentially unlisted telephone numbers. All the telephone interviews were conducted between 6–10:30 pm to avoid over-sampling non-working individuals. Unanswered telephone calls were given at least three attempts before being classified as invalid. Unavailable eligible participants were contacted again. If necessary, appointments were made. All the participants were briefed by the trained interviewers. Verbal informed consent was sought from the participants prior to commencement of the interviews; the interviewers signed a form pledging having completed the required consent procedures. The anonymous interview took 10 to 15 min to complete. No incentives were given to the participants. The response rates (i.e., the number of completed interviews divided by the number of eligible respondents contacted) of the two surveys were 56.2% and 56.8%, respectively.

### Measurements

#### Background information

Socio-demographics were collected, including sex, age, educational level, current marital status, and whether being told by doctors as having any of the listed chronic diseases (hypertension, diabetes, chronic pulmonary diseases, heart stroke, heart failure, cerebrovascular diseases, Alzheimer’s disease, ulcerative diseases, liver diseases, and tumors).

#### Probable depression

It was assessed by the 9-item Patient Health Questionnaire (PHQ-9), which was validated in Chinese populations [26]. The items asked about the frequencies that specific depressive symptoms had occurred during the past 2 weeks. Sample items involved those related to “little interest or pleasure in doing things” and “feeling down, depressed, or hopeless” (4-point Likert scales: 0 = not at all to 3 = nearly every day). Summative scores ≥ 5, 10, 15, and 20 were defined as having mild, moderate, moderately severe, and severe depression, respectively. The Cronbach’s alphas were 0.84 and 0.79 in the two surveys, respectively. Following many studies [27, 28], a binary variable of probable (moderate or above) depression, defined as summative PHQ-9 score ≥ 10, was formed.

#### Resource losses related to COVID-19

The 16-item CORS-COVID-19 has been satisfactorily validated in the Chinese general population in Hong Kong, China [20]. It consists of the five domains of resource losses due to COVID-19, including loss in financial resource (e.g., “reduced incomes”), loss in fun (e.g., “loss of fun in life”), loss in future control (e.g., “more difficult to control one’s future”), loss in social resource (e.g., “reduced social support from friends”), and loss in family resource (e.g., “less communication among family members”). The items were rated by using 3-point Likert scales (1 = no loss at all to 3 = loss to a great extent); higher mean scores indicated higher levels of resource losses. The Cronbach’s alpha of the five subscales ranged from 0.77 to 0.92 and 0.72 to 0.92 in the two surveys, respectively.

### Study design

Like other surveillance studies, two serial cross-sectional surveys were conducted, using the same questionnaire. Under this design, the data of the two serial surveys were pooled into a whole sample. The time of survey (Round 2 versus Round 1) was used as the binary independent variable and probable depression as the dependent variable; resource losses were tested as mediating variables.

### Sample size planning

Sample size planning was conducted by using the Tests for Two Proportions [Differences] module of the PASS 11.0 [NCSS LLC, Kaysville, United States of America (USA)]. The sample size of 200 in Round 1 and 450 in Round 2 allowed us to detect the smallest difference of 0.067 between the two groups (alpha = 0.05; power = 0.80, two-sided).

### Statistical analysis

Chi-square test and *t*-test were conducted to test the between-group differences in categorical and continuous variables, respectively. Multivariable logistic regression models were performed to test significance of the associations between the key variables [i.e., survey time (Round 2 versus Round 1) and the five domains of resource losses] and probable depression. The interaction effects were tested by fitting two sets of hierarchical logistic regression models containing a pair of main effect variables (e.g., survey time and loss in financial resource) with and without the interaction term (e.g., survey time × loss in financial resource); statistical significance was tested by comparing the two models’ goodness of fit [Δ-2 Log Likelihood (Δ-2LL)]. Background variables were adjusted for; adjusted odds ratios (a*OR*) and the corresponding 95% confidence intervals (*CI*s) were presented. Pearson correlation coefficients were used to examine correlations between scores of CORS-COVID-19 and PHQ-9. To test mediation effects, structural equation modeling (SEM) using the weighted least square mean and variance adjusted estimator was fit. A latent variable involving the five subscales of the COR-COVID-19 was fit. The recommended levels of model fit indices included *χ*^*2*^*/df* ≤ 5, Comparative Fit Index (CFI) ≥ 0.90, and Root Mean Square Error of Approximation (RMSEA) ≤ 0.80. The bootstrapping method (*n* = 2000) was performed to test the existence of the indirect effect, which was indicated by the 95% *CI*s of indirect effect not including zero. SPSS 23.0 (IBM Corp., Armonk, New York, USA) and Mplus 7.0 (Muthén & Muthén, Los Angela, USA) were used to perform data analysis. Statistical significance was defined as two-tailed *P*-value < 0.05.

## Results

### Participants’ characteristics

The mean age was 45.5 years [standard deviation (*SD*) = 13.6; range: 21.5–65.5]; over half were female (59.7%). About one-third had attained an educational level of college or above (32.6%) and were currently married (31.9%); 20.5% self-reported having at least one chronic disease. These factors were adjusted for in subsequent analyses. The samples of the two rounds of survey showed no statistically significant differences in these characteristics (Table 1).

**Table 1:** Characteristics of the two samples

	Overall	Round 1	Round 2	*P of Chi-square*
	*n* (%)	*n* (%)	*n* (%)	
Overall	667	209	458	
Age (Mean, *SD*)^†^	45.5, 13.6	46.7, 13.0	45.0, 13.8	0.124
Sex				
Female	398 (59.7)	125 (59.8)	273 (59.6)	0.961
Male	269 (40.3)	84 (40.2)	185 (40.4)	
Educational level^¶^				
< College	442 (67.4)	129 (62.6)	313 (69.6)	0.079
≥ College	214 (32.6)	77 (37.4)	137 (30.4)	
Marital status^¶^				
Others	453 (68.1)	139 (66.5)	314 (68.9)	0.546
Married	212 (31.9)	70 (33.5)	142 (31.1)	
Chronic disease status^¶^				
No	530 (79.5)	169 (80.9)	361 (78.8)	0.545
Yes	137 (20.5)	40 (19.1)	97 (21.2)	
Probable depression^¶^				
None-to-mild (PHQ-9 < 10)	603 (96.5)	191 (91.4)	412 (99.0)	< 0.001
Moderate-to-severe (PHQ-9 ≥ 10)	22 (3.5)	18 (8.6)	4 (1.0)	

*SD*: Standard deviation; PHQ-9: The 9-item Patient Health Questionnaire. ^†^*t*-test was conducted to test the between-group difference. ^¶^Missing data was excluded from data analyses (less than 2%)

### Changes in CORS-COVID-19 and PHQ-9 scores over time

The prevalence of the two surveys was 12.0% versus 5.3% for mild depression, 6.2% versus 1.0% for moderate depression, and 2.4% and 0% for severe depression (*P* < 0.001; Additional file 1: Table S1). The prevalence of probable depression (defined as PHQ-9 ≥ 10) reduced significantly from 8.6% at Round 1 to 1.0% at Round 2 (Table 1). Similarly, the mean PHQ-9 score decreased significantly from 2.6 (*SD* = 4.0; range: 0–21) to 0.8 (*SD* = 1.9; range: 0–14); the effect size of the difference was moderate-to-strong (Cohen’s *d* = 0.55) (see Table 2). In Table 2, except for the domain of loss in fun (mean = 2.3 at Round 1 versus mean = 2.2 at Round 2; Cohen’s *d* = 0.07), the overall scale of the CORS-COVID-19 and all the four other domains of resource losses also reduced significantly and substantially from Round 1 to Round 2. The moderate to strong effect sizes (Cohen’s *d*) regarding changes in financial, social, and overall losses over time were 0.88, 0.60, and 0.56, respectively, and were small to moderate for losses in family and future control (0.36 and 0.39, respectively).

**Table 2:** Between-group differences in the multidimensional resource losses and PHQ-9 score between the two surveys

	Range	Overall	Round 1	Round 2	*P* of *t*-test	Cohen’s *d*
		Mean,* SD*	Mean, *SD*	Mean, *SD*		
Resource loss of the CORS-COVID-19						
Loss in financial resource	1–3	1.5, 0.6	1.9, 0.7	1.4, 0.5	< 0.001	0.88
Loss in fun	1–3	2.2, 0.6	2.3, 0.6	2.2, 0.5	0.332	0.07
Loss in future control	1–3	1.7, 0.7	1.9, 0.7	1.6, 0.7	< 0.001	0.39
Loss in social resource	1–3	1.6, 0.5	1.8, 0.6	1.5, 0.4	< 0.001	0.60
Loss in family resource	1–3	1.1, 0.3	1.2, 0.4	1.1, 0.3	< 0.001	0.36
Overall scale	1–3	1.6, 0.4	1.8, 0.4	1.6, 0.3	< 0.001	0.56
Depressive symptoms (PHQ-9 score)	0–21	1.4, 2.9	2.6, 4.0	0.8, 1.9	< 0.001	0.55

*SD*: Standard deviation; CORS-COVID-19: Conservation of Resource Scale for COVID-19; PHQ-9: The 9-item Patient Health Questionnaire. Cohen’s *d* values of 0.20‒0.49, 0.50‒0.79, and ≥ 0.80 refer to small, moderate, and large effect size, respectively

### Adjusted associations between resource losses and probable depression

Among all the participants, the overall scale and subscale scores of the CORS-COVID-19 were all significantly and positively associated with probable depression (Table 3). All the interaction effects between the CORS-COVID-19 scores and survey time on probable depression were statistically non-significant (*P* > 0.05; see Additional file 1: Table S2).

**Table 3:** Adjusted associations between resource losses and probable depression

	Probable depression	
	a*OR* (95% *CI*)	*P*
Resource loss of CORS-COVID-19		
Loss in financial resource	6.31 (3.19‒12.50)	< 0.001
Loss in fun	2.72 (1.15‒6.42)	0.022
Loss in future control	5.59 (2.74‒11.40)	< 0.001
Loss in social resource	9.96 (4.46‒22.22)	< 0.001
Loss in family resource	10.48 (1.88‒22.50)	< 0.001
Overall scale score	42.30 (13.41‒133.44)	< 0.001

DV: Dependent variable; a*OR*: Adjusted odds ratio; *CI*: Confidence interval; CORS-COVID-19: Conservation of Resource Scale for COVID-19. The models were adjusted for sex, age (years), educational level, marital status, and chronic disease status

### Bivariate correlations

Within the pooled sample, the overall scale and subscale scores of CORS-COVID-19, which were significantly correlated with each other (*r* ranged from 0.15 to 0.80, *P* < 0.001), were also positively correlated with PHQ-9 score (*r* ranged from 0.14 to 0.42, *P* < 0.001) (see Table 4). Within the two survey samples, similar patterns of correlations were found; the results are not shown in the tables.

**Table 4:** Bivariate correlations among resource losses and PHQ-9 scores

	1	2	3	4	5	6
1. Loss in financial resource	–					
2. Loss in fun	0.27***	–				
3. Loss in future control	0.53***	0.41***	–			
4. Loss in social resource	0.34***	0.25***	0.23***	–		
5. Loss in family resource	0.30***	0.15***	0.22***	0.40***	–	
6. Overall scale score of the CORS-COVID-19	0.80***	0.62***	0.77***	0.61***	0.51***	–
7. PHQ-9 score	0.31***	0.14***	0.33***	0.30***	0.39***	0.42***

CORS-COVID-19: Conservation of Resource Scale for COVID-19; PHQ-9: The 9-item Patient Health Questionnaire. ***P < 0.001

### SEM

**Fig. 1:**
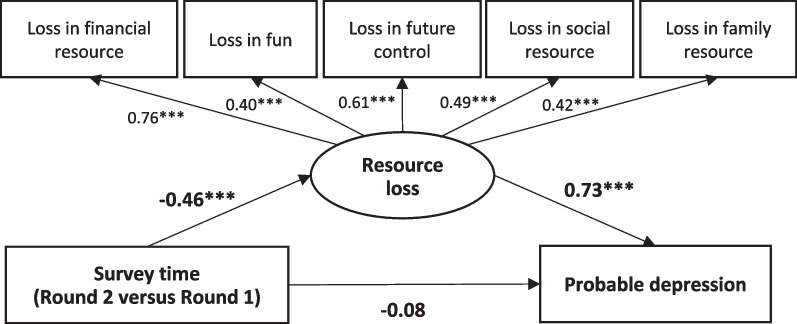
Structural equation modeling testing the mediation effect of resource loss (a latent variable) between survey time and probable depression (standardized coefficients were reported; ***P < 0.001)

The model fit index of the measurement model of the latent variable of resource loss was satisfactory after the addition of two error covariance terms (between loss in family/social resource and between loss in future control/fun) (χ^2^/*df* = 98.77/33 = 3.0 < 5; CFI = 0.90, RMSEA = 0.06). The structural mediation model in Fig. 1 also showed satisfactory model fit index (χ^2^/*df* = 98.77/33 = 3.0 < 5; CFI = 0.90, RMSEA = 0.06). It is seen that survey time [Round 2 versus Round 1 (the reference group)] was negatively associated with the latent variable of resource loss (*β* = − 0.46), i.e., the level of overall resource loss reduced from Round 1 to Round 2. The latent variable of resource loss was in turn positively associated with probable depression (*β* = 0.73). The indirect mediation effect between survey time and probable depression via the latent variable of resource loss was statistically significant (*β* = − 0.34, 95% *CI*: -0.45 to -0.23). As the direct effect of survey time on probable depression was non-significant (*β* = − 0.08, 95% *CI*: -0.26–0.11), the aforementioned indirect effect was a full mediation. The SEM model was repeated by using the summative score of PHQ-9 as a continuous dependent variable; a highly consistent full mediation effect was found (see Additional file 1: Figure S1).

## Discussion

The prevalence of probable depression of the Hong Kong Chinese adult general population observed at Round 1 was higher than that of a survey conducted in 2009 (8.6% versus 4.3%) [29], although lower than the 11.2% reported in another local survey conducted in 2019. These surveys used the same tool and cut-off point (i.e., PHQ-9 ≥ 10). This study observed a substantial decline in the prevalence of probable depression over time during the pandemic, corroborating two empirical studies conducted in the United Kingdom (UK) [5, 6].

Hong Kong, China was adversely affected by the pandemic. At Round 1, high levels of unemployment rate, social distancing control (e.g., restriction of gathering size), and perceived risk of COVID-19 infection [30] were observed. Comparing Round 1 with Round 2, all except one type of resource losses had reduced moderately to strongly. Financial loss became smaller as the economy was recovering at Round 2. As social distancing was associated with lower social interactions and social support [31] and was relaxed at Round 2, the level of social resources increased. Improvements in the epidemiology and economy might create hope and lead to increase in future control. In both rounds, the loss of fun was the largest among all types of resource loss and remained stable over time. This construct involved an item regarding outbound traveling. Recreational traveling is a source of ‘fun’ to many people of Hong Kong, China. There were 302 million travelers crossing the Hong Kong boundary in 2019 versus only 3500 on May 31, 2021. Throughout the entire study periods, it was difficult for Hong Kong people to travel to the mainland of China and abroad as it required mandatory quarantine and frequent testing. The policy has recently been relaxed by some countries. In addition, Hong Kong people might worry about infection as a high number of new COVID-19 infections were recorded in some common destinations (e.g., a peak of 300,000 and 68,000 new cases per day in the USA and the UK, respectively). The lack of improvement in traveling might partially explain why there was no gain in the construct of ‘fun’.

In this study, all types of resource loss were significantly associated with depression. Specifically, the association between losses in family and social resources showed stronger effects on probable depression than the other types of resource losses. Interpersonal relationships (e.g., social support) were important determinants of depression during the pandemic [32]. According to Stress Coping Theories, supportive social relationship is an important coping resource buffering negative impacts of stressors on mental health problems [33]. Consistent with the literature [34], the associations between losses in financial resource/future control and probable depression are understandable as financial burdens and worries about future are serious stressors. Having fun is important in maintaining a good mood [35]; literature has shown that boredom was positively associated with mental distress amid the COVID-19 pandemic [36].

To our knowledge, this study was the first one explaining reduction in depression by the reduction in resource losses due to COVID-19 over time during the pandemic. This is understandable, as there were fewer resource losses over time, while resource losses were positively associated with probable depression. Longitudinal studies are warranted to consolidate the observed mediation effect. Policy makers need to balance between control and resource losses and be made aware that interventions reducing resource losses, such as temporary financial assistance and employment, organization of online recreational activities, and tangible and intangible support for social/family relationships, might alleviate population mental distress over time. Some pilot randomized controlled studies modifying such factors are warranted.

This study’s data were collected in April 2020 and May 2021, respectively, i.e., in an early phase of the pandemic. The timing needs discussion as the pandemic has been changing. During May 2021 to January 2022, the pandemic was under relatively good control in Hong Kong, China; no new local COVID-19 cases were detected for 88 days (October 2021 to early January 2022). As mentioned, relatively mild social distancing measures were implemented. However, the fifth wave hit Hong Kong, China from late January 2022 to the present (February 2023), with daily new infections increased from 92 on January 31 to the peak of about 56,000 on March 3, 2022. The government then closely adhered to the zero-COVID-19 policy [37] and strict control measures were implemented, including short-term locking up of housing blocks reporting COVID-19 infections for compulsory testing, banning incoming flights, 21-day/14-day/7-day quarantine for people entering Hong Kong (till December 2022), suspension of classes, vaccination proof for entry to public venues, restrictions on restaurant gathering size, banning household visits, and more. The number of cases was then brought down to < 1000 a day from April 15 to June 15, 2022. Since September 2021, the government apparently started moving away from the zero COVID policy although there were still around 3000–5000 new cases per day. It is uncertain whether and how much resource losses and depression have been caused by this fifth wave, but according to our findings, it is inferred the levels would be substantial. New factors of depression may have emerged during the later phase of the pandemic. For instance, a Hong Kong study found prevalent ‘burnt out’ regarding COVID-19 prevention [38], which seems to be a very interesting and global phenomenon. Future studies may look at its impacts on depression and the relationship with resource losses (e.g., loss in fun). Overall, although this study presented changes in an earlier phase, its findings are still implicative as resource losses are general in nature and common from the earlier to later and even the end of pandemic phases.

This serial study has important potential implications. Comparative surveillance studies are needed to investigate changes in depression over time. Globally, depression is prevalent during the COVID-19 pandemic [2]; the urgency to reduce depression during the pandemic has been emphasized by World Health Organization (WHO) [39]. Factors of depression included various perceptions [40], emotions and fear [41], financial stress [42], and interpersonal relationships [43]. Importantly, the strengths of the factors of depression are not stagnant but instead, vary according to the context (e.g., epidemiology and strictness of control measures). Although many countries are lifting their control measures, the pandemic has not ended. Furthermore, mutations to new severe strains and new pandemics may emerge. Depression at population level is thus still a concern at present and in the future. Understanding changes in an early phase of the pandemic would inform interventions and policy making to reduce depression at later phases and when new pandemic emerges.

This study also contributes to theory development. One of the strengths is that it was based on the important COR theory and a validated scale of resource losses. A majority of the global population has lost some resources throughout the pandemic; the losses are ongoing as recovery is only preliminary. Resource losses determined mental distress related to traumatic events [13, 14], similar associations were found in this study. The associations and the significant mediation gave support to the COR theory, which postulates that resource losses would result in negative mental health outcomes [8]. Interestingly, the strength of the observed associations involving resource losses did not vary over time, suggesting that such factors and the model of this study are relevant in various phases of the pandemic, including the recovery phase. A third time point may be added to this study to inspect longer term relationships. As the COVID-19 pandemic was a brand-new experience, it is important to document changes at all phases. Hence, although the data was collected in 2020/2021, the findings remain implicative in 2022 and beyond.

This study has some methodological limitations. First, causal or temporal inferences cannot be made due to the serial cross-sectional design, which has commonly been used to compare prevalence at two time points within the same population to denote changes in the population [44]. The two samples (Round 1 and Round 2) came from the ‘same’ general population, as the change in the composition of this large Hong Kong population over a few months was negligible. It is analogous to taking two snapshots and a dramatic change in the prevalence of probable depression (from 8.6% to 1.0%) was observed. This design may be weaker than the longitudinal design, as it was able to detect changes at only the population level but not individual level; future longitudinal studies are warranted to confirm the findings. Second, social desirability bias may exist as people may be less likely to report depressive symptoms to avoid potential stigma. Third, the characteristics of the participants and refusers may differ, although the response rate of this study (56.2% and 56.8%) was comparable to those of other local telephone surveys. The upper limit of the age range of the sample was 70 years because older people might have different perceptions about resource losses, such as financial arrangements and control over the future. Fourth, the sample size in this study, although acceptable, was only moderate. Yet, the difference in the prevalence of probable depression between the two rounds was 0.076, which was larger than the smallest detectable difference in proportion. A retrospective power analysis suggests a statistical power of 0.89 if the population proportions were the same as those obtained from this study (0.086 and 0.010). Published papers using similar survey designs have involved comparable sample size [45, 46]. The sample size was thus adequate. Fifth, selection bias may exist, as only about 80% of the people in Hong Kong have a fixed-line phone at home [47]. Nonetheless, the study sample may be reasonably representative of the Hong Kong general population as simple random sampling was used. The sample’s mean age was 46.7/45 years at Round 1/Round 2 versus 46.4 years of the Hong Kong Census data (2021). Males seemed to have been under-estimated as the percentage was 40.2%/40.4% at Round 1/Round 2 but was 47.4% in census. Sixth, generalization of the findings to other countries/populations needs to be made cautiously, as Hong Kong may have her specific context.

## Conclusions

This study observed a significant reduction in the prevalence of probable depression in the adult general population in Hong Kong, China over a 12-month period during the COVID-19 pandemic, together with significant substantial declines in all but one of the domains of resource losses. The exception was the dimension loss in fun. Furthermore, the reduction in resource losses fully explained the reduction in probable depression over time. This novel finding emphasizes the importance of reducing mental health problems via reducing various types of resource losses. This study gives support to the COR theory in understanding changes in mental distress associated with an impactful pandemic. Importantly, it is not necessary that the general public would become and remain depressed during the entire course of the COVID-19 pandemic. Instead, the situation is highly malleable. It is essential that the government, health workers, and all stakeholders (e.g., employer and employee) work together to restore mental health at the coming phases of the pandemic. Longitudinal and cross-countries studies are useful to confirm the predictive effects of changes in resource losses on depression. On-going surveillance is warranted to keep track of the dynamic changes in various aspects of resource losses and prevalence of depression in the general and special populations. Data obtained at an early phase may inform later phases of COVID-19 pandemic and new pandemics. Lastly, the resource losses are unlikely to be evenly distributed across populations within a country. It is possible that disadvantaged groups might have disproportionately high levels of resource loss, and hence worse mental distress and slower remissions. The inequity issues about resource losses and their relationships with mental distress due to COVID-19 need to become a focus of future studies.

## Supplementary Information

**Additional file 1.** **Table S1.** Prevalence of probable depression of the two samples. **Table S2.** Testing the interaction effects betweenthe variables of resource losses and survey time onto probable depression.

## Data Availability

The data was available on reasonable request.

## Abbreviations

COVID-19: Coronavirus disease 2019
COR: Conservation of Resource Theory
PHQ-9: The 9-item Patient Health Questionnaire
CORS-COVID-19: Conservation of Resources Scale for COVID-19
SEM: Structural equation modeling
a*OR*: Adjusted odds ratio
HKD: Hong Kong Dollar
USD: United States Dollar
GDP: Gross domestic product
USA: United States of America
*CI*: Confidence interval
CFI: Comparative fit index
RMSEA: Root mean square error of approximation
*SD*: Standard deviation
UK: United Kingdom
WHO: World Health Organization
