# Supplementary material for: Reduction in COVID-19 related resource loss and decline in prevalence of probable depression in Chinese adults: an application of the Conservation of Resource Theory
Source: Infect Dis Poverty. 2023 Mar 16;12:19. doi: 10.1186/s40249-023-01068-1 (PMC10018587; doi:10.1186/s40249-023-01068-1)
Supplement: Supplementary file 1 — Additional file 1. Table S1. Prevalence of probable depression of the two samples. Table S2. Testing the interaction effects betweenthe variables of resource losses and survey time onto probable depression. [file 40249_2023_1068_MOESM1_ESM.docx]

Table 1 Prevalence of probable depression of the two samples

|  | Overall | Round 1 | Round 2 | *P of Chi-square* |
| --- | --- | --- | --- | --- |
|  | *n* (%) | *n* (%) | *n* (%) |  |
| Overall | 667 | 209 | 458 |  |
| Probable depression ^¶^ |  |  |  | < 0.001 |
| None (PHQ-9 < 5) | 556 (89.0) | 166 (79.4) | 390 (93.8) |  |
| Mild (PHQ-9 = 5–9) | 47 (7.5) | 25 (12.0) | 22 (5.3) |  |
| Moderate (PHQ-9 = 10–14) | 17 (2.7) | 13 (6.2) | 4 (1.0) |  |
| Severe (PHQ-9 ≥ 15) | 5 (0.8) | 5 (2.4) | 0 (0.0) |  |

PHQ-9: The 9-item Patient Health Questionnaire. ¶, Missing data was excluded from data analyses (less than 2%).

Table S2 Testing the interaction effects between the variables of resource losses and survey time onto probable depression

|  | Probable depression | | | |
| --- | --- | --- | --- | --- |
|  | a*OR* (95% *CI*) | *P* | a*OR* (95% *CI*) | *P* |
| Loss in financial resource | 4.57 (2.17–9.63) | < 0.001 | 3.49 (1.52–8.01) | 0.003 |
| Survey time | 0.20 (0.06–0.65) | 0.008 | 0.02 (0.00–1.26) | 0.065 |
| Loss in financial resource × Survey time |  |  | 2.78 (0.52–14.92) | 0.233 |
| Δ-2LL | 1.501 | | | |
|  |  |  |  |  |
| Loss in fun | 2.23 (0.95–5.26) | 0.066 | 1.99 (0.78–5.07) | 0.150 |
| Survey time | 0.10 (0.03–0.32) | < 0.001 | 0.02 (0.00–7.60) | 0.201 |
| Loss in fun × Survey time |  |  | 1.85 (0.20–17.49) | 0.590 |
| Δ-2LL | 0.304 | | | |
|  |  |  |  |  |
| Loss in future control | 4.94 (2.29–10.64) | < 0.001 | 6.78 (2.59–17.76) | < 0.001 |
| Survey time | 0.13 (0.04–0.41) | 0.001 | 1.39 (0.03–59.04) | 0.864 |
| Loss in future control × Survey time |  |  | 0.36 (0.07–1.80) | 0.215 |
| Δ-2LL | 1.509 | | | |
|  |  |  |  |  |
| Loss in social resource | 6.89 (2.91–16.27) | < 0.001 | 4.74 (1.87–12.07) | 0.001 |
| Survey time | 0.20 (0.06–0.63) | 0.006 | 0.01 (0.00–0.76) | 0.038 |
| Loss in social resource × Survey time |  |  | 4.76 (0.65–35.01) | 0.125 |
| Δ-2LL | 2.494 | | | |
|  |  |  |  |  |
| Loss in family resource | 7.51 (3.35–16.83) | < 0.001 | 5.86 (2.52–13.66) | < 0.001 |
| Survey time | 0.15 (0.05–0.47) | 0.001 | 0.02 (0.01–0.45) | 0.015 |
| Loss in family resource × Survey time |  |  | 4.21 (0.62–28.61) | 0.141 |
| Δ-2LL | 2.912 | | | |
|  |  |  |  |  |
| Overall resource loss | 28.60 (8.44–96.92) | < 0.001 | 20.70 (5.29–81.06) | < 0.001 |
| Survey time | 0.25 (0.08–0.82) | 0.021 | 0.02 (0.00–8.43) | 0.199 |
| Overall resource loss × Survey time |  |  | 3.47 (0.22–55.46) | 0.378 |
| Δ-2LL | 0.816 | | | |

a*OR*: Adjusted odds ratio; *CI* = Confidence interval; Δ-2LL = Δ-2 Log Likelihood. The models were adjusted for sex, age (years), educational level, marital status, and chronic disease status.

**Survey time**

**(T2 versus T1)**

Loss in financial resource

Loss in fun

Loss in future control

Loss in social resource

Loss in family resource

**Depressive symptoms**

**-0.52*****

**0.60*****

**0.02**

0.66***

0.31***

0.52***

0.52***

0.49***

**Indirect effect: *β* = -0.31, 95% *CI*: -0.42**–**-0.21**

**Model fit index: χ^2^/*df* = 128.69/29 = 4.4 < 5; CFI = 0.90; RMSEA = 0.07**

**-0.08**

Figure S1 Structural equation modeling testing the mediation effect of resource loss (a latent variable) between survey time and depressive symptoms (standardized coefficients were reported; ***, *P* < 0.001; *CI* = Confidence interval; CFI = Comparative Fit Index; RMSEA = Root mean square error of approximation)
